# Radical resection of a giant retroperitoneal calcifying fibrous tumor combined with right hepatectomy and reconstruction of the inferior vena cava and bilateral renal veins

**DOI:** 10.1186/s40792-018-0417-4

**Published:** 2018-01-18

**Authors:** Masaki Kimura, Hiroki Kato, Seishiro Sekino, Narihiro Ishida, Katsutoshi Murase, Katsuya Shimabukuro, Takafumi Sekino, Kiyoshi Doi, Masayuki Matsuo

**Affiliations:** 1 Department of General and Cardiothoracic Surgery, Graduate School of Medicine, Gifu University, 1-1 Yanagido, Gifu, 501-1194 Japan; 2 Department of Radiology, Graduate School of Medicine, Gifu University, Gifu, Japan

**Keywords:** Calcifying fibrous tumor, Retroperitoneal tumor, Radical resection, IVC reconstruction, Renal vein reconstruction

## Abstract

**Background:**

We report a case of a giant retroperitoneal calcifying fibrous tumor (CFT) treated by radical tumor resection combined with right hepatectomy and reconstruction of the inferior vena cava (IVC) and bilateral renal veins. Only three case reports of CFT arising in the retroperitoneum have been reported until today.

**Case presentation:**

In a 19-year-old female patient, computed tomography (CT) images showed a well-demarcated expansile lesion around the IVC accompanied by focal calcification, whereas the IVC that was circumferentially surrounded by the lesion was dilated due to the desmoplastic reaction. On magnetic resonance imaging (MRI), the lesion demonstrated heterogeneous hypointensity on T2-weighted images. Delayed enhancement was observed on dynamic contrast-enhanced CT and MRI. ^18^F-Fluorodeoxyglucose (FDG) positron emission tomography (PET)/CT images showed increased FDG uptake [maximum standardized uptake values (SUVmax), early image 7.28; delayed image 7.40]. On operative examination, because the tumor adhered to the liver parenchyma, the right Glisson capsule, and the origin of bilateral renal veins, radical tumor resection combined with right hepatectomy and reconstruction of the IVC and bilateral renal veins was performed.

**Conclusions:**

Radical tumor resection was successfully and appropriately performed for a young patient with a giant retroperitoneal CFT with a view to achieving complete venous reconstruction and safe surgical margins for a potentially malignant tumor.

## Background

Calcifying fibrous tumor (CFT) is a rare benign mesenchymal tumor characterized by a hypocellular fibroblastic proliferation with associated chronic inflammation and variably prominent calcification. CFT has been described in superficial and deep somatic soft tissues, such as the upper and lower extremities, neck, scrotum, back, and abdominal wall, as well as in intracavitary and visceral locations, such as the gastrointestinal tract, mesentery, mediastinum, omentum, peritoneum, and pleura. Our literature search was performed by using PubMed as the search engine. When we entered the keyword “retroperitoneal calcifying fibrous tumor” or “retroperitoneal calcifying fibrous pseudotumor” in the search field of the initial screen, we could retrieve six relevant articles. Among them, only three case reports of CFT arising in the retroperitoneum have been reported so far [1–3]. A case of a giant retroperitoneal CFT, which circumferentially surrounded the dilated inferior vena cava (IVC), treated by radical tumor resection combined with right hepatectomy and reconstruction of the IVC and bilateral renal veins is reported.

## Case presentation

A 19-year-old woman presented with common cold symptoms to a primary care physician. Her chief complaints were cough and fever. She had previously been healthy with no relevant past or family history. The doctor regarded her as having a common cold and treated her with medication for a common cold, but she did not recover. A subsequent computed tomography (CT) examination showed right pneumonia and a giant retroperitoneal tumor, and she was referred to our hospital.

**Fig. 1:**
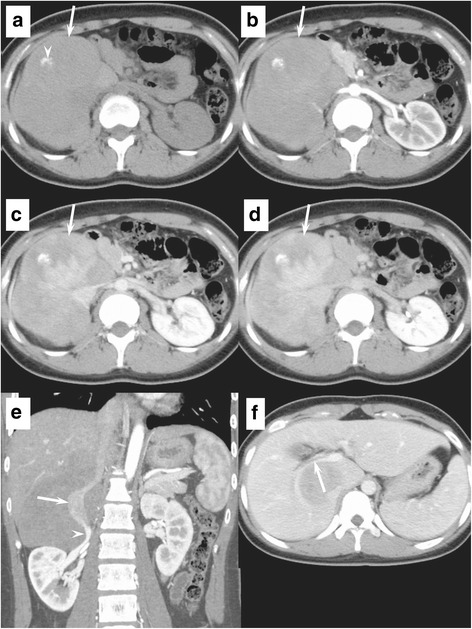
Abdominal computed tomography. **a** Unenhanced CT image shows a well-demarcated, expansile, soft tissue lesion (arrow) with focal calcification (arrow head) in the right peritoneum. **b**–**d** Dynamic contrast-enhanced CT images show delayed enhancement (arrows). **e** Coronal reformatted contrast-enhanced CT image shows dilatation of the IVC lumen circumferentially surrounded by the lesion (arrow) and stenosis of the right renal vein (arrow head). **f** Contrast-enhanced CT image shows the compressed right Glisson capsule (arrow)

**Fig. 2:**
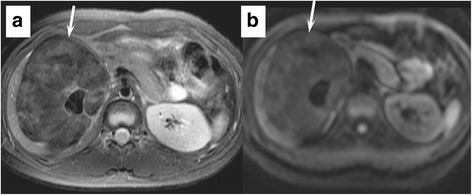
Abdominal magnetic resonance imaging. **a** Fat-suppressed T2-weighted fast spin-echo image (TR/TE, 1600/80 ms) shows a heterogeneously hypointense lesion, suggestive of abundant fibrosis (arrow). **b** Diffusion-weighted echo-planar image (TR/TE, 2291/47 ms) shows heterogeneous hypointensity without strong diffusion restriction (ADC value = 1.39 × 10^−3^ mm^2^/s) (arrow)

Physical examination showed a hard lump under her right rib cage. Her conjunctivae were neither anemic nor icteric. On admission, liver function tests and tumor markers (CEA, AFP, and CA19-9) were within normal ranges. She was classified as Child–Pugh class A, and her indocyanine green retention rate at 15 min (ICGR15) was 2.1%. The right diaphragm was elevated on chest X-ray examination. CT images showed a 118 × 99 × 114 mm^3^, well-demarcated, expansile lesion located between the liver and right kidney (Fig. 1a–d). Focal calcification was found within the lesion (Fig. 1a). It enhanced gradually from the arterial phase to the equilibrium phase on dynamic contrast-enhanced CT (Fig. 1b–d). The IVC below the IVC–hepatic vein junction was circumferentially surrounded by the lesion, whereas the IVC lumen was dilated (Fig. 1e). Stenoses of the origins of bilateral renal veins were observed (Fig. 1e). The right Glisson capsule was compressed, and mild dilatation of the intrahepatic biliary ducts was observed in the posterior segment of the right lobe (Fig. 1f). Although the main feeding artery was a branch of the right adrenal artery, the normal right adrenal gland was demonstrated adjacent to the lesion. The fat layer was confirmed between the lesion and other adjacent organs (right kidney, duodenum, and pancreas). On magnetic resonance imaging (MRI), fat-suppressed T2-weighted images showed a heterogeneously hypointense lesion, suggestive of abundant fibrosis (Fig. 2a). Diffusion-weighted images showed heterogeneous hypointensity without strong diffusion restriction [apparent diffusion coefficient (ADC) value = 1.39 × 10^−3^ mm^2^/s] (Fig. 2b). Delayed enhancement was also observed on dynamic contrast-enhanced MRI. On ^18^F-fluorodeoxyglucose (FDG) positron emission tomography (PET)/CT images, the maximum standardized uptake value (SUVmax) of the lesion was 7.28 in the early phase and 7.40 in the delayed phase. No other abnormal uptake was noted. Endoscopic examination of the whole digestive tract found no other digestive system abnormalities. Intermediate or malignant fibroblastic/myofibroblastic tumors, such as desmoid tumor, solitary fibrous tumor, inflammatory myofibroblastic tumor, or fibrosarcoma, were considered as preoperative differential diagnoses.

**Fig. 3:**
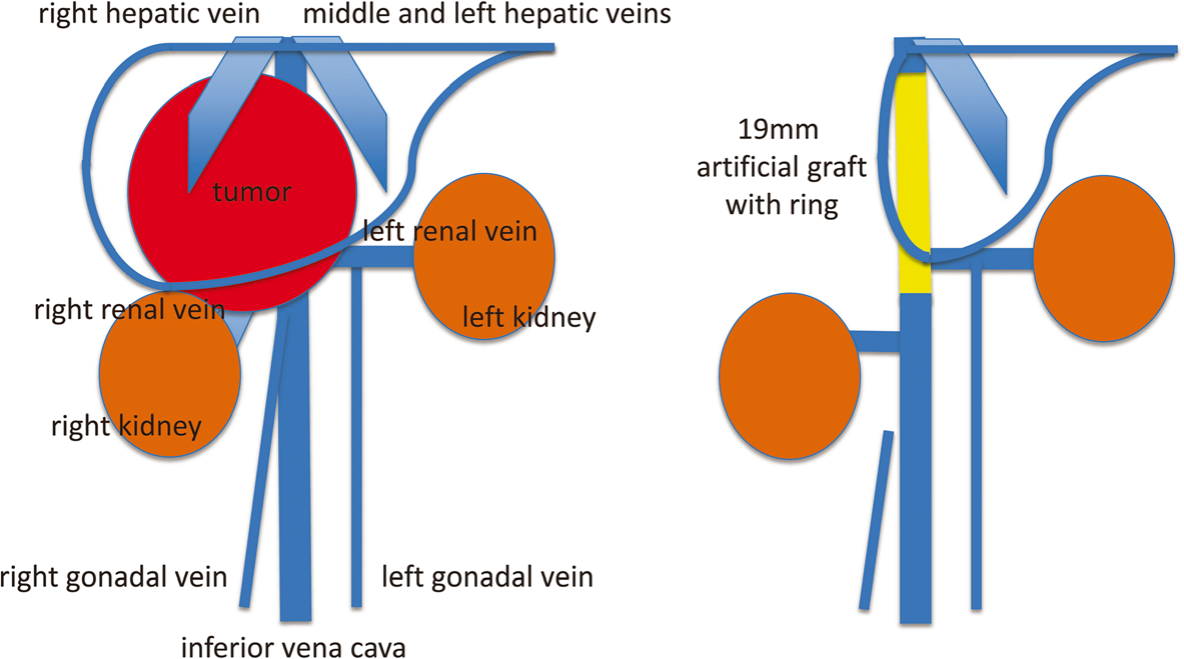
Schema of the preoperative state (left) and postoperative state (right). Surgical procedures of venous reconstruction are as follows: (1) right gonadal vein ligation, (2) right renal vein reconstruction by suturing to the native IVC, (3) IVC reconstruction replaced by the artificial graft, and (4) left renal vein reconstruction by suturing to the artificial graft

Radical tumor resection combined with right hepatectomy and reconstruction of the IVC and bilateral renal veins was planned. An incision was placed below the right hypochondrium that extended to the left rectus abdominis muscle and xiphoid process. Although the giant retroperitoneal tumor could be exfoliated from the pancreas, duodenum, and right kidney, it was difficult to peel off the tumor from the liver parenchyma, Glisson capsule of the posterior segment of the right lobe, and right adrenal gland. The IVC was circumferentially surrounded by the tumor from below the origin of the hepatic vein to the origins of the renal veins. The origins of bilateral renal veins were also involved by the tumor, but bilateral renal arteries were intact. After the right renal vein was reconstructed by suturing to the native IVC, right hepatectomy and right adrenalectomy were then performed. Because the blood pressure did not drop with IVC clamping, the tumor was removed en bloc with the right liver, the right adrenal grand, the IVC, and the origins of the bilateral renal veins. The IVC was replaced by a 19-mm artificial graft with ring, and the left renal vein was reconstructed by suturing to the artificial graft (Fig. 3). Total operative time was 575 min, and total blood loss was 3150 g. Blood transfusion of 4 RBC-LR units was required during the operation.

**Fig. 4:**
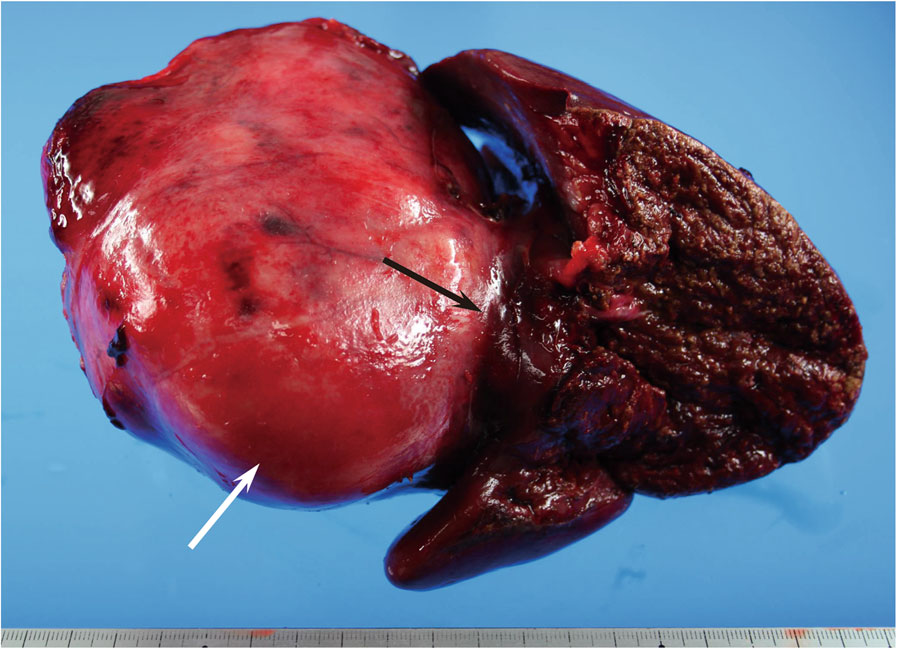
Resected specimen. The resected tumor (white arrow) has a lobulated contour and white-colored surface. The tumor tenaciously adheres to the Glisson capsule (black arrow)

**Fig. 5:**
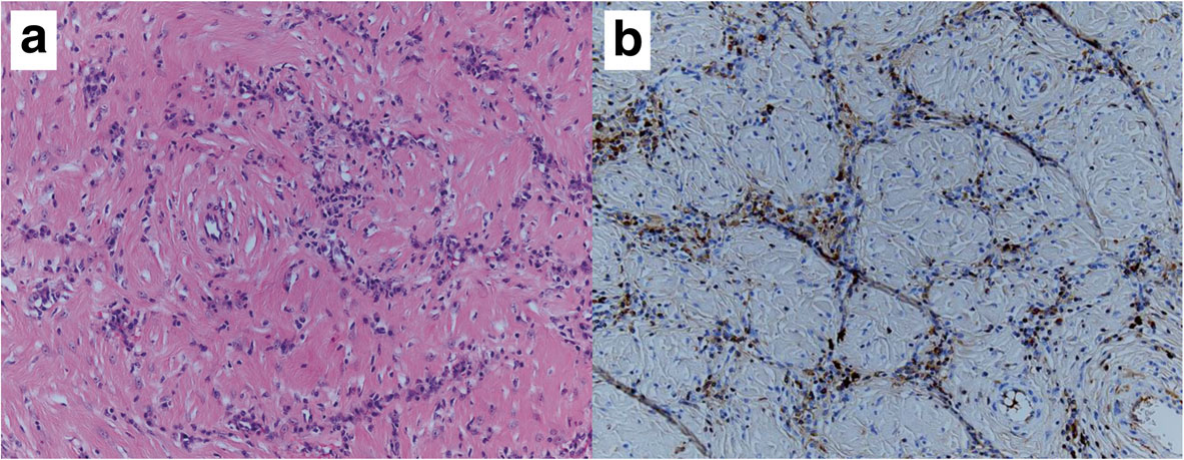
Histological findings and immunohistochemical studies. **a** H&E stain (× 20) shows a whorled bundle of collagen fiber and hyalinized stroma with hypocellular spindle cells. Infiltration of inflammatory cells is observed along small vessels. **b** IgG4 immunohistochemical stain (× 20) shows numerous IgG4-positive plasma cells

The resected tumor was lobulated and elastic hard, and the surface was white-colored (Fig. 4). On histopathological examination, a whorled bundle of collagen fiber and hyalinized stroma with hypocellular spindle cells was seen. Infiltration of inflammatory cells, such as plasma cells and lymphocytes, was observed along small vessels (Fig. 5a). Scattered psammomatous calcification was confirmed. Abnormal vascular proliferation was found within the IVC wall, whereas tumor cell invasion into the IVC wall was not confirmed. Both IgG and IgG4 immunostains were positive (Fig. 5b). The histopathological diagnosis was CFT. The patient’s postoperative course was generally good, and she was discharged from hospital on postoperative day 11. More than a year and a half after the operation, the reconstructed IVC and bilateral renal veins were patent. At present, no residual tumor or tumor recurrence has been observed. Compensatory hypertrophy of the residual liver parenchyma is adequate, and liver function test results are within normal limits.

### Discussion

CFT has also been referred to as childhood fibrous tumor with psammoma bodies or calcifying fibrous pseudotumor in the previous medical literature [4, 5]. The term CFT was established by the classification of soft tissue and bone tumors of the World Health Organization (WHO) in 2002. According to a review article of 157 patients with CFT, the mean age of patients was 33.58 years, and the ratio between men and women was 1:1.27 [6]. The mean diameter of the tumors was 4.6 cm, ranging from 0.1 to 25 cm, and larger tumors appeared in the neck and adrenal gland [6]. The percentage of asymptomatic patients was 30.57% [6]. Local recurrences occur in a subset of patients, and recurrences may occur repeatedly [7].

Ultrasonographically, CFT is a well-circumscribed mass with varied echogenicity accompanied with an acoustic shadow because of scattered calcification. CT is superior to ultrasound for the detection of calcification. CT images demonstrated a well-circumscribed soft tissue mass with various degrees of calcification [6, 8]. On MRI, CFT is characterized by hypointensity on T1- and T2-weighted images, reflecting abundant collagen fiber and hyalinized stroma [6, 8]. A delayed enhancement pattern is observed on dynamic contrast-enhanced imaging [8]. The present case also showed these characteristic imaging findings, and the IVC was circumferentially surrounded by the CFT, whereas the IVC lumen was dilated. The IVC dilatation caused by the desmoplastic reaction may be one of the characteristic imaging findings of CFT. The imaging findings of retroperitoneal CFT are summarized in Table 1.

**Table 1:** The imaging findings of retroperitoneal calcifying fibrous tumor

	Size (cm)	US	CT	MRI	PET (SUVmax)
Acenero et al. [1]	13	NA	Homogeneously enhanced mass with focal calcification	NA	NA
Jaiswal et al. [2]	20 × 15	Well-defined hetero-echoic mass	Heterogeneously enhanced mass	NA	NA
Prochaska et al. [3]	6 × 6 × 5	NA	Homogeneously enhanced mass with focal calcification	NA	NA
Present case	12 × 10 × 11	NA	Heterogeneously enhanced mass with focal calcification	Heterogeneously hypointense mass on T2-weighted images	7.28

*US* ultrasound sonography, *CT* computed tomography, *MRI* magnetic resonance imaging, *PET* positron emission tomography, *SUVmax* maximum standardized uptake value, *NA* not available

Safe surgical margins needed to be ensured, because the preoperative differential diagnoses included intermediate or malignant fibroblastic/myofibroblastic tumors. Because the tumor could not be peeled off the Glisson capsule and right adrenal gland, right hepatectomy and right adrenalectomy in association with radical tumor resection were necessary. Avoidance of excessive organ resection and complete reconstruction of the venous circulation were required in this operation, whereas excessive resection of the other adjacent organs, such as the duodenum, pancreas, and right kidney, was avoided. Until today, postoperative compensatory hypertrophy of the residual liver parenchyma is adequate and liver and adrenal gland dysfunction has not been observed. We believe that a minimally invasive operation could be achieved.

One of the greatest achievements in the present case was renal function preservation. Because the need to reconstruct both the IVC and bilateral renal veins was expected, an extracorporeal circulation circuit for the blood pressure reductions caused by clamping the IVC below the liver was prepared [9, 10]. First, the right renal vein was reconstructed by suturing to the native IVC. Second, after right hepatectomy and right adrenalectomy, the IVC below the IVC–hepatic vein junction was resected and replaced by a 19-mm artificial graft with ring. Finally, the left renal vein was reconstructed by suturing to the artificial graft. Therefore, the bilateral kidneys were protected from ischemia or congestion during the operation, and postoperative renal function was preserved.

## Conclusions

Radical tumor resection was successfully and appropriately performed for a young patient with a giant retroperitoneal CFT with a view to achieving complete venous reconstruction and safe surgical margins for a potentially malignant tumor.

## Abbreviations

ADC: Apparent diffusion coefficient
CFT: Calcifying fibrous tumor
CT: Computed tomography
FDG: Fluorodeoxyglucose
ICGR15: Indocyanine green retention rate at 15 min
IVC: Inferior vena cava
MRI: Magnetic resonance imaging
PET: Positron emission tomography
SUVmax: Maximum standardized uptake values
WHO: World Health Organization
